# Traumatic injury among females: does gender matter?

**DOI:** 10.1186/1752-2897-8-8

**Published:** 2014-07-28

**Authors:** Ayman El-Menyar, Hany El-Hennawy, Hassan Al-Thani, Mohammad Asim, Husham Abdelrahman, Ahmad Zarour, Ashok Parchani, Ruben Peralta, Rifat Latifi

**Affiliations:** 1 Clinical Research, Trauma Surgery Section, Hamad General Hospital, PO Box 3050, Doha, Qatar; 2 Clinical Medicine, Weill Cornell Medical School, Doha, Qatar; 3 Internal Medicine, Ahmed Maher Teaching Hospital, Cairo, Egypt; 4 Trauma Surgery Section, Hamad General Hospital, Doha, Qatar; 5 Department of Surgery, Arizona University, Tucson, AZ, USA

**Keywords:** Trauma, Gender, Injury mechanisms, Female

## Abstract

**Background:**

Trauma remains one of the leading causes of morbidity and mortality worldwide. Generally, the incidence of traumatic injuries is disproportionately high in males. However, trauma in females is underreported.

**Aim:**

To study the epidemiology and outcome of different mechanisms and types of traumatic injuries in women.

**Methods:**

We conducted a traditional narrative review using PubMed, MEDLINE and EMBASE, searching for English-language publications for gender-specific trauma between January 1993 and January 2013 using key words “trauma”, “gender”, “female” and “women”.

**Results:**

Among 1150 retrieved articles, 71 articles were relevant over 20 years. Although it is an important public health problem, traumatic injuries among females remain under-reported.

**Conclusion:**

There is a need for further research and evaluation of the exact burden of traumatic injuries among females together with the implementation of effective community based preventive programs.

## Introduction

Traumatic injuries are considered a significant burden on healthcare system worldwide. According to National Trauma Institute in US, traumatic injuries accounted for 30% of all life years lost in the 2009 [1]. The effect of traumatic injuries on life years lost is equivalent to the life years lost from cancer, cardiovascular disease (CVD) and HIV together. Trauma is the third leading cause of death among all age groups and the mortality rate is exceptionally high particularly among young age group [1]. Moreover, the annual incidence of burn-related mortalities exceeds 300,000 worldwide [2]. A recent report from Qatar showed that the burden of diseases among men was found three times more than of women's [3]. For men, chronic diseases like CVD (15.7%) and motor vehicle crashes (MVCs) (13.7%) represent a great burden and an important source of lost years of healthy life. Generally, women are under-representative in all kind of studies including non-trauma medical disorders due to socio-cultural barriers [4-6]. However, women sustaining traumatic injuries should have true representation of the disease burden, as it needs acute interventions. Despite that, there is a lack of relevant literature on the incidence of trauma involving female gender. We reviewed all the relevant studies from our institute during the last 5 years period (Table 1) [3,7-18]. In these studies, representation of females was very low in comparison to males. Does this observation represent the true care provided to females and does it influence the healthcare approach to and the strategy for females? We do not have a clear answer.

**Table 1:** Studies from our institute during the last 5 years (traumatic and non-traumatic)

**Authors**	**Studies**	**Female/Male**
**El-Menyar et al. [**[7]**]**	Chest injury	6%/94%
**AbdulRahman et al. [**[8]**]**	Multiple rib fracture	7.3%/92.7%
**El-Menyar et al. [**[9]**]**	Traumatic abdominal injury	7%/93%
**Faramawy et al. [**[10]**]**	Traumatic spinal injury	8%/92%
**Moamena et al. [**[11]**]**	Traumatic head injury	8%/92%
**Abdulrazzaq et al. [**[12]**]**	Pedestrians injury	8.1%/91.9%
**Zarour et al. [**[13]**]**	Diaphragmatic injury	16%/84%
**Tuma et al. [**[14]**]**	Fall from height	0%/100%
**Atique et al. [**[15]**]**	Fall of heavy objects	3%/97%
**Bener et al. [**[3]**]**	Measuring burden of diseases	24.5%/75.5%
**Latifi et al. [**[16]**]**	Pedestrian injuries	7%/93%
**El-Menyar et al. [**[17]**]**	Acute Coronary Syndrome	24%/76%
**Al-Thani et al. [**[18]**]**	Peripheral arterial disease	35%/65%

To focus on the incidence, characteristics, mechanism, risk factors, outcome and preventive strategies for trauma in females, we conduct a traditional narrative review of the literature utilizing the search engines: PubMed, MEDLINE and EMBASE by using key words “trauma”, “gender”, “female”, and “women” between January 1993 and January 2013. We excluded abstracts and non-English articles. The review included trauma regardless the severity, mechanism of injury, hospital complications and outcomes. Among 1150 articles, 71 articles were relevant. The majority of articles were traumatic brain injury- followed by pregnancy –related trauma. Therefore, although it is an important public health problem, traumatic injuries among females remain under-reported.

### Incidence and mechanism of traumatic injuries

#### Motor Vehicle Crash (MVC)

The annual incidence of MVC-related mortality is about 1.24 million worldwide [19]. More than three-quarters (77%) of all road traffic deaths occur among men. Also, the fatality rate increased three-folds among young males (<25 years of age) compared to young females [19]. This could be explained by greater exposure of driving and patterns of high risk behavior in males [20,21]. In many developed countries, there is high incidence of pedestrian mortality among males. According to WHO report, males involving in pedestrian fatalities accounted for 70% and 61% in US and Singapore, respectively [22]. Among child pedestrians involving MVC, boys are usually involved in more incidents than young girls. This is because boys are more likely than girls to cross roads unaccompanied by an adult [22]. Traffic collisions involving children on bicycles report higher fatality rates for boys. The incidence of mortality was two times higher for boys using bicycles compared to girls [22]. In elderly population, the rate of mortality from MVC was two-three folds higher in males than elderly females in developing countries [22]. With respect to MVC-related injuries, women are at a greater risk of injuries to lower extremities particularly ankle/tarsal injuries due to smaller stature [22]. According to a recent report from US, female occupants (aged 20-35 yrs) of motor vehicles were 28-31% more susceptible for fatal injuries than males from similar crash due to smaller body stature [22].

Further, gender differences might impact the social and economic consequences of injury-related temporary and/or permanent disabilities. Women may typically not be in jobs that have an adequate insurance coverage or allow for long duration of absence from work. They may not be able to pay for home-based nursing care, and for childcare and paid domestic help that may be needed because of their temporary or permanent impairment. On the other hand, as men are often the sole income earners in many families, injury-related disabilities may affect the overall household economy to a greater extent [22].

Notably, in most of the gulf region, driving license is difficult for females and this may in part explain the lower rate of MVCs among females. Moreover, the traditional female dressing in that region of the world may explain in part the pedestrian injuries in females.

#### Gender and fall

**Figure 1:**
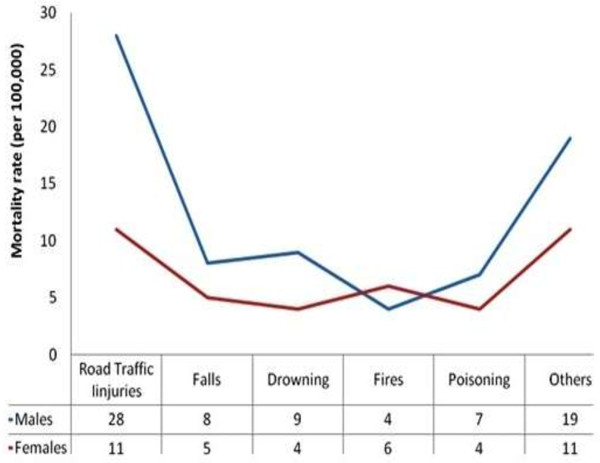
**Global mortality by gender from unintentional injury.** Modified from WHO: *Noncommunicable Disease and Mental Health Cluster* ref [24].

Falls are a serious public health concern worldwide. It is the second major cause of fatal unintentional injuries, subsequent to MVCs [23]. In particular, a higher frequency (80%) of fall-related deaths has been reported from developing countries. The highest rate of fall-related mortality is observed among older age group (>60 yrs) [23]. Generally, men and women are at equal risk of fall related injuries, irrespective of age groups and regions (Figure 1). However, fall-related mortality rates are highest among males in the low and middle-income countries [24]. Whereas, the incidence of nonfatal falls-related injuries is more common in women compared to men [25]. Older women and younger children are more susceptible to falls and sustained severe injuries [23]. However, higher levels of risk-taking behaviors and occupational hazards are the key factors associated with increased mortality and DALYs among males [23]. Further, falls may be associated with fracture. Among the US community-dwelling elderly, low-impact falls lead to half a million osteoporotic fractures per year [26]. The major risk factors for fracture included age, female gender, menopause before age of 45 and visual impairment [27]. Kantayaporn et al. [27] reported visual impairment in elderly women is highly associated with fall-related fractures. Also, in comparison to men, older women had two times higher rates of fall-related fractures [28]. The majority (95%) of hip fractures are caused by falls [29]. According to National Center for Health Statistics, hip fractures in women are three times higher than men. Also black women have significantly lower risk of hip fracture compared to white women [30]. An increased risk of fall is also observed in relation to living environment such as slippery floor in the house and bathroom/toilet located outside the house [31]. Contrarily, elderly people living with spouse had a 32% lesser chance of experiencing a fall compared to those who lived alone [31]. Stevens & Dellinger showed a significant increase in fall-related death rates among whites in US, with an annual increase of 3.6% for men and 3.2% for women, respectively [32].

#### Gender and burn

Burns remains serious public health concern which accounts for an annual mortality of 310,000, globally in 2004 [33]. Low- and middle-income countries represents majority of these fatalities (4.8 per 100,000 per year). It is evident that women and young children are at greater risk of domestic burns. Several studies have reported female gender to be at high risk of mortality in burn injuries [34-36]. Also, females are at high risk for the development of hypertrophic scarring after burn injury [37].

In the acute phase of burn injuries, pediatric females have reasonably higher levels of anabolic hormones than the male patients, thereby sustaining decreased hypermetabolism and need shorter intensive care unit stay [38]. In another subsequent study, Jeschke et al. [39] showed that females have shorter intensive care units (ICUs) and hospital stay than males after severe thermal injury. This is because, females who sustained burns exert an attenuated inflammatory and hypermetabolic response than males. Better ICU prognosis in females with improved muscle protein net balance, preserved lean body mass and reduced resting energy expenditure prediction was reported in females when compared to males [38].

A study based on a large population of burn injury patients (34,470 men and 14,609 women) showed that women had a 50% increased risk of death when compared to men. Also, age stratification showed higher risk of mortality among females of all age groups between 10 and 70 years [40]. In contrast, Barrow et al. and Kobayashi et al. found no significant difference in mortality rate by genders among severely burned children and adults, respectively [41,42]. McGwin et al. [43] concluded that a significant correlation was observed between mortality and gender with respect to age. Mortality rate among females of <60 years was over twice that of males, however, no difference was noted among those > 60 years. These associations persisted following adjustment for potentially confounders. Therefore, women of <60 years of age with burn injuries have an increased risk of death compared with males.

### Trauma in pregnancy

In the United States, trauma is the leading cause of maternal morbidity and mortality [44]. Trauma accounted for most of the non-obstetric mortalities among young women of 14 and 44 years in the study by Mighty [45]. The incidence of traumatic injury varies from 6% to 7% among pregnant women [46] and most injuries are minor, therefore the rate of hospitalization is only 0.3% to 0.4% [47]. Pregnancy is associated with a number of physiological and anatomical changes, that may be expected to increase the morbidity and mortality among women who sustain injury during pregnancy [48,49]. However, the influence of hormone levels had considerable effect on outcome in post trauma pregnant women. John et al. [44] observed lower mortality among pregnant women in comparison to the females of the same age groups with equal injury severity. This study suggested a possible role of hormonal and physiologic status during pregnancy in improved trauma outcomes [44].

Mendez-Figueroa et al. [50] found that domestic violence, partner violence and MVC are the predominant causes of reported trauma during pregnancy [50]. Similarly, Shah et al. [51] stated that around 70% of injuries in pregnant women are related to MVCs followed by due to domestic violence (12%). George et al. [52] found high injury severity scoring (ISS) and abdominal abbreviated injury score (AIS), longer hospital and intensive care LOS, high incidence of intubation and placenta abruption to be significantly associated with unsuccessful pregnancy. The reported incidence of placental abruption is 1% to 5% in minor injuries and 20% to 50% in major injuries, with all abruptions occurring within 6 hours of trauma [53,54]. Women delivering at the trauma hospitalization had the worst outcomes, regardless the severity of the injury and should be monitored closely during the subsequent pregnancy [55]. Management of trauma during pregnancy should be focused on maternal stabilization which depends upon severity of traumatic injuries [50]. The study of Scorpio et al. [56] showed fetal outcome is significantly associated with ISS and admission serum bicarbonate levels of the pregnant women after trauma. Moreover, fetal outcome is not affected by surgery or diagnostic peritoneal lavage and these procedures should not be delayed, if indicated [56].

### Trauma in elderly women

Falls remains the major **c**ause of non-fatal injuries in geriatric trauma with progressively increasing rates of mortality. Over a period of ten years, the mortality among geriatric falls raised to 45.3% and 59.5% in men and women, respectively [57]. Fractures, open wounds, contusions, sprains/strains, and traumatic brain injury (TBI) are the frequently observed injuries in falls. Especially, older women are more prone to trauma due to co-morbidities, such as osteoporosis. Osteoporosis severely affects older women who are at high risk of hip fracture [58]. The incidence of hip fractures is disproportionately higher in women than men [29]. The medical cost in management of these injuries is high due to the need of surgery, hospitalization, and extensive rehabilitation [59]. Moreover, women with chronic medical conditions or functional impairments are at higher risk of subsequent traumatic injuries [60]. However, in US, according to NHTSA report, the incidence of elderly women involving in MVCs is equivalent to the elderly men [61].

### Females and fragility fractures

Due to the decrease in oestrogen production after the menopause, females are at a greater risk of osteoporosis than males [62]. The number of females with osteoporosis increases from 2% at age 50 to > 25% at age 80 [62]. Osteoporosis is often asymptomatic in its early stage until fracture happens. Fragility fractures are fractures that result from mechanical forces (low energy trauma) that would not result in fracture under normal situation [63]. Fragility fractures are associated with substantial disability, reduced quality of life and excess mortality in older people [62].

Hip fracture, a common example of fragility fractures, always requires hospitalization and is fatal in 20% of patients. Moreover, it permanently causes disabilities in 50% of affected patients as only 30% of patients fully recover [63]. The frequency of low energy pelvic fracture admissions in patients > 50 years old has been shown to increase by 58% and 111% in males and females, respectively [64]. An audit from Ireland showed that females accounted for 75% of hip fractures that on average occur 7 years earlier in comparison to males [62]. The 1-month mortality was 7%, whereas 1-year mortality was 22.6% for females and 27.8% for males [62]. For a long time, the majority of fragility pelvic fractures were managed conservatively and prior studies showed a lower mortality and morbidity with early aggressive surgical management of neck of femur fractures when compared with delayed or conservative management [64]. It has been noted that the outcomes are similar regardless of the displacement or fracture type [64]. However, there are no randomized comparative studies comparing operative to conservative management in fragility fractures of the pelvis.

### Females and home trauma

According to the Monash University Accident Research Centre [65], home is the second location, after transport, of fatal injuries to adult females accounting for 45% of home injury hospital admissions (HIHA). Also, sixty-eight percent of hospital admission related to home injury constituted elderly women (>60 years).

Falls cause the majority of HIHA followed by intentional self-inflicted injury. The peak times for home injuries are from Saturday to Wednesday (inclusive) and in the summer months. According to Victorian Coroners’ Facilitation System data, 56% of reported home injury adult female deaths are intentional. Women aged >55 years had 1.5 times increased risk of all-cause mortality compared to young women in house-hold injuries [65].

### Gender differences and trauma pathophysiology

Several studies have identified gender as a factor influencing complication rates and outcomes after traumatic injury [66-69]. The possible explanation for this heterogeneity is the differential effects of sex steroids such as anabolic or catabolic steroids [70,71]. Therefore, post injury pathogenesis varies significantly according to gender [72]. There is also difference in the physiological response to injury between men and women. Houston-Bolze et al. [73] reported an increase in the insulin-like growth factor (IGF)-1 concentration and little change in transthyretin levels with the increase in the severity of injury in women. In contrast, the levels of IGF-1 and transthyretin decrease with increasing injury severity among men. Another study by Ertan et al. [74] found significantly higher levels of plasma interleukin-1beta levels among male with trauma hemorrhage than female patients. Also, post-trauma, female patients had significantly shorter ICU stay than males because of higher anabolic hormones levels, which causes decreased proinflammatory response and hypermetabolism [75]. Moreover, there is a gender-specific variability among blood glucose levels in trauma patients which could be used a predictor of outcome. Mohr et al. [76], reported an increased association between blood glucose variability and mortality rate among males. A study based on major burn trauma, showed that post-injury myocardial function and myocardial inflammatory responses are gender-specific [77].

### Gender differences in trauma immunopathology

Traumatic injuries patients generally had suppressed immune functions due to severe hemorrhage. Moreover, young men, ovariectomized and elderly women are reported to have severe immunosuppression [78]. However, females sustaining traumatic hemorrhage in premenopausal age have maintained immune functions and better prognosis. Therefore, it is evident that sex hormones are crucial for maintaining the host immune response in trauma patients. Besides cytokine production, immune cells also express receptors for androgen and estrogen. Therefore, cytokine production is modulated by local production of active steroids in immune cells [78]. Choudhry et al. [72] described beneficial effect of female sex hormone on immunomodulation. Whereas, male sex steroids suppresses immune and cardiac functions in hemorrhagic trauma patients. A study based on animal model showed gender-specific suppression of cell-mediated immune response after burn injury [79]. The investigators reported lower T-helper 1 cytokine production in males than in female mice. These findings suggest that the level of steroid hormones at the time of injury correlates well with the outcome of trauma.

### Gender and outcome

Some studies analyzing traumatic injuries have identified gender-specific differences in the outcome [80]. George et al. [81] found a significant association between male patients of increased age (>50 yrs) and mortality after blunt trauma. Mostafa et al. [80] observed higher incidence of multiple organ failure, longer intensive care unit and hospital LOS, and higher mortality in young males in comparison to females. Napolitano et al. [68] found no association between gender and mortality in blunt trauma patients who did not develop pneumonia. Moreover, patients who developed pneumonia had 2.8 to 5.6 fold increased risk of mortality among females compared to males. Rappold et al. [82] also found no protective effect of female gender on the development of ARDS, pneumonia, sepsis, or lower mortality after blunt trauma. In burn trauma patients, the risk of mortality was doubled among women aged 30-59 years than in men of similar age group [83]. Trauma related to low falls exhibited greater survival benefits with respect to pre-existing conditions and male gender [84].

Studies have suggested differential response of nervous system towards traumatic brain injury (TBI) in male and female gender. According to Ottochian et al. [67], female gender and increased age (≥55 yrs) are independent predictors of mortality in isolated severe TBI patients. Kraus et al. [85] reported a hospital mortality difference of 28% between females and males as multivariate analysis demonstrated that females were 1.75 times more likely to die than males. Farace and colleagues [86] conducted a meta-analysis and reported worse outcomes for females. On the other hand, Leitgeb et al. [87] could not find any effect of gender on outcomes after TBI. Moreover, functional outcome in head injury patients lacks significant correlation between male and female gender [88].

### Strategies for prevention of traumatic injury among women

Strategies focusing gender-specific risk factors are imperative for injury prevention. Awareness campaigns for safer driving and working conditions should be launched publically. School-age children and adolescents of both genders should be targeted. Older men and women are highly susceptible for trauma, therefore, supportive environment for geriatric populations is needed during in-door and out-door activities. Injury prevention programmes should focus on building positive behavioral change in the community through public awareness and educational events. Planning rehabilitation services for trauma patients should consider gender differences in the socioeconomic consequences of temporary and/or permanent disability post injury.

Prevention of domestic falls especially among elderly women should be emphasized through effective fall prevention programs which include creating safer environments, education and training in geriatric population [23]. Community based fall prevention programs should include identification and management of potential risk factors, such as screening for living environments; clinical interventions to identify risk factors, like treatment of low blood pressure, Vitamin D and calcium supplementation, treatment of correctable visual impairment. Home assessment and environmental modification are essential for individuals with known risk factors or a history of falling. Prescription of appropriate assistive devices to address physical and sensory impairments; muscle strengthening and balance retraining prescribed by a trained health professional are needed for community-based group programs. Mass counseling regarding safety, proper clothing to be worn at time of cooking and first aid measures are essential for burn injury prevention.

It is recommended to improve the safety of all road users. However, special focus should be given to improve car and street designs for more safety and visibility. In addition, educational programs should improve the safety of older people in road traffic [89].

### Summary & conclusion

There is considerable effect of gender on the traumatic injury outcome. This could be explained by the anatomical, physiological, immunological and hormonal differences which play an important role in the gender-specific trauma outcome. In almost all types and mechanism of injuries, the frequency of male gender is disproportionately high and masks the impact of trauma in female population [90,91]. Further research should emphasize the main causes of Injury, differences in treatment, complications, and long term outcomes among females. So, there is a need of extensive research for evaluation of exact magnitude of traumatic injuries in women together with effective implementation of community based preventive interventional programs.

## Competing interests

The authors declare that they have no competing interests.

## Authors’ contributions

AE carried out data analysis and interpretation, drafted and reviewed the manuscript. HE participated in data analysis, interpretation, and helped to draft the manuscript. HA was involved in study design, data collection and review of the manuscript. MA participated in data analysis, coordination and helped to draft the manuscript. HA conceived of the study, participated in its design and review of the manuscript. AZ participated in the study design, data interpretation, and helped to review the manuscript. AP involved in the study design, data interpretation, and drafted the manuscript. RP participated in study design, data interpretation, and helped to draft the manuscript. RL conceived of the study, and participated in its design, data interpretation, and reviewed the manuscript. All authors read and approved the final manuscript.
